# Decreased concentrations of intracellular signaling proteins in colon cancer patients with BRAF mutations

**DOI:** 10.1038/s41598-020-77109-8

**Published:** 2020-11-18

**Authors:** Dorte Aa. Olsen, Caroline E. B. Thomsen, Rikke F. Andersen, Jonna S. Madsen, Anders Jakobsen, Ivan Brandslund

**Affiliations:** 1 Department of Biochemistry and Immunology, Lillebaelt Hospital, University Hospital of Southern Denmark, Beriderbakken 4, 7100 Vejle, Denmark; 2 Department of Oncology, Lillebaelt Hospital, University Hospital of Southern Denmark, Vejle, Denmark; 3 Department of Regional Health Research, University of Southern Denmark, Odense, Denmark; 4 Danish Colorectal Cancer Center South, Lillebaelt Hospital, University Hospital of Southern Denmark, Vejle, Denmark

**Keywords:** Biochemistry, Cancer

## Abstract

The activation of intracellular signaling pathways plays a critical role in cancer pathogenesis. The current study aims to quantify intracellular signaling proteins in localized colon cancer tissue to investigate the prognostic value of these biomarkers and elucidate their possible relations to mutation status. Colon cancer tissue and autologous reference tissue were collected from 176 patients who underwent colon cancer surgery. Assays were developed to quantify ERK, AKT and cyclin d using single-molecule array technology. KRAS/BRAF/PIK3CA mutation status was determined using droplet digital PCR. Patients with BRAF mutations had decreased concentrations of ERK (p = 0.0003), AKT (p = 0.0001) and cyclin d (p = 0.003), while no significant differences were found between patients with KRAS mutations and wild-type patients. None of the investigated proteins were associated with disease-free survival or overall survival when all patients were included. However, when patients were stratified according to mutation status, significant correlations with overall survival were seen for patients with BRAF mutations and AKT (p = 0.002) or ERK (p = 0.03) and for KRAS mutations and cyclin d (p = 0.01). Conclusions: A strong correlation exists between intracellular signaling protein concentrations and mutational BRAF status. Overall survival in colon cancer patients depends on both gene mutation status and signaling protein concentrations.

## Introduction

**Figure 1:**
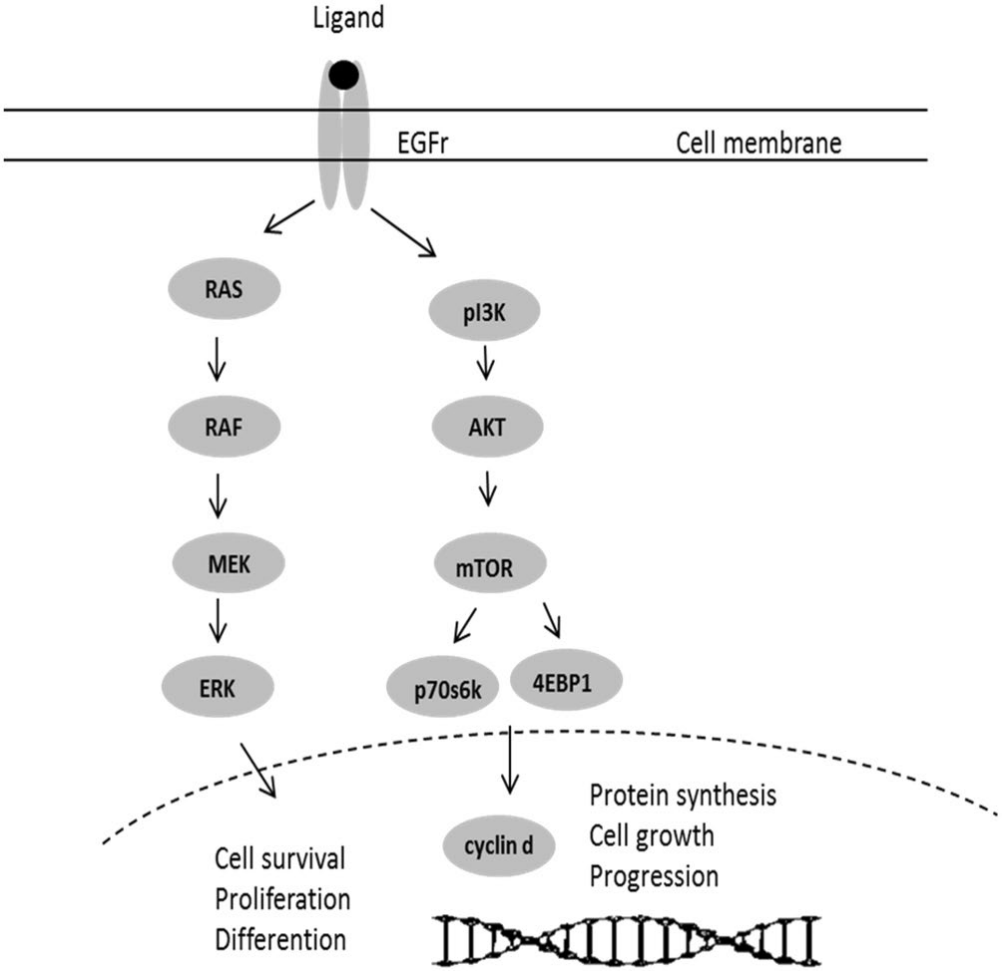
Simplified graphic illustration of the EGFr pathways RAS/RAF/MEK/ERK and PI3K/AKT.

The intracellular signaling network of the epidermal growth factor receptor (EGFR) consists of two key signal pathways: the mitogen-activated protein kinase (MAPK), also termed RAS/RAF/MEK/ERK, and the phosphatidylinositol 3-kinase (PI3K)/ protein kinase B (AKT) pathways. These signal pathways interact in a complex and coordinated manner to regulate all stimulated cellular processes and have been described in detail^1,2^. Both ERK and AKT activate more than 100 downstream proteins from the cytosol to the nucleus, including transcription factors, protein kinases, phosphatases and cytoskeletal elements. Thus, they are involved in a wide variety of nuclear and cytosolic processes, including cell differentiation and proliferation and oncogenic transformation^3–5^. A schematic overview of the pathways is presented in Fig. 1.

The activation and dysregulation of intracellular signaling pathways plays a critical role in cancer. Alterations in RAS and RAF proteins are frequently seen in colorectal cancer and result in constitutively active proteins that stimulate the ERK signaling pathway even though no signal is present. The occurrence of KRAS and BRAF mutations in colorectal cancers is approximately 40% and 10–25%, respectively^6–8^. Moreover, dysregulation of the PI3K/AKT pathway due to the activation of mutations in PI3K (PIK3CA) has been identified in colorectal cancer^9–11^ , and PIK3CA mutations have been found to coexist with KRAS or BRAF mutations^10^.

The use of inhibitors against growth factor receptors and tyrosine kinase activators has become standard anti-cancer therapy during the last 10–20 years. Some of the monoclonal antibodies used in the treatment are Cetuximab as a blocker to EGFR in colorectal cancer and the Trastuzumab HER2 receptor blocker in breast cancer. However, mutations in the receptor or pathway proteins result in resistance to monoclonal antibody treatment. Therefore, it is important to detect such mutations at an early, pre-treatment phase to predict whether patients will benefit from the treatment or only experience its side effects.

Known mutations are usually detected by PCR or sequencing. Though these methods are routinely performed in the laboratory, they tend to be more laborious and expensive compared to protein-based methods, and they often cause a delay of several days for reporting. In addition, only currently known mutations will be detected and as the number of known clinically relevant mutations increases, so will the expense for sequencing or detecting mutations by PCR. Protein-based methods might, therefore, serve as an alternative method of reflecting mutation status and/or dysregulations within intracellular signaling pathway proteins.

To enable testing of the clinical impact of these proteins, we developed quantitative protein assays for measuring phosphorylated ERK (pERK) as a marker of MAPK pathway activation and phosphorylated AKT (pAKT) for PI13K/AKT pathway activation. Moreover, we established methods for measuring the total protein levels of ERK (tERK), AKT (tAKT), and cyclin d.

## Materials and methods

### Patients

The study included 176 patients who underwent colon cancer surgery at Vejle Hospital from 2010–2013. Baseline patient characteristics including age, gender, pT-category, stage, nodal metastasis and differentiation grade are shown in Table 1. Four patients had no available clinical data. All patients gave written informed consent to participate in the study, which was approved by the Ethics Committee for Southern Denmark (S-20140178). All methods were performed in accordance with the relevant guidelines and regulations.

**Table 1:** Baseline patient characteristics (n = 172).

Parameters		n (%)
Age years, median (range)	79 (47–98)	
Gender	Male	83 (48)
	Female	89 (52)
pT-category	T1	0 (0)
	T2	11 (6)
	T3	138 (80)
	T4	23 (14)
Stage	I	8 (5)
	lI	96 (56)
	III	68 (39)
Nodal metastasis	N0	104 (60)
	N1	68 (40)
Differentiation grade	High	141 (82)
	Low	17 (10)
	Mucinous	14 (8)

*N* number, *pT-category* pathologic tumor stage.

### Reagents

Capture antibodies tAKT and pAKT (DYC887B) (R&D Systems, Minneapolis, MN, USA), tERK and pERK (DYC1230C) (R&D Systems), and cyclin d (ab218793, Abcam, Cambridge, UK) were covalently attached by standard carbodiimide coupling chemistry to carboxylated paramagnetic beads (Quanterix, Billerica, MA, USA). The biotinylated detector antibodies and the calibrators were tAKT (DYC1775), pAKT (DYC887B), tERK (DYC1230C), pERK (DYC1018B) (R&D Systems), and cyclin d (ab218793) (abcam). Streptavidin-β-galactosidase (SβG), enzyme substrate resorufin-β-d-galactopyranoside (RGP) and all consumables including wash buffers, cuvettes, disposable tips, and discs were from Quanterix.

### Single molecule array (Simoa)

To quantify AKT, ERK and cyclin d, single-plex assays were developed for the automated Simoa HD-1 Analyzer platform (Quanterix). Details about the methods are described in Supplementary Information S1. Colon cancer tissue was dissected along with autologous reference tissue by an experienced pathologist. The tissue was stored in RNAlater (Qiagen, Hilden, Germany) at − 20 °C until use. Colon cancer tissue and autologous reference tissue were homogenized in lysis buffer (50 mM Tris–HCl, 150 mM NaCl, pH 7.5, 1% triton X-100), including protease and phosphatase inhibitor cocktail 10 µl/ml lysis buffer using the Dispomix system (Xiril, Hombrechtikon, Switzerland). The samples were then centrifuged at 16,000*g* at 4 °C for 15 min and the supernatant was recovered, aliquoted and stored at − 80 °C until use. Total protein concentration was measured using the BCA protein assay reagent (Pierce, Rockford, IL, USA). The tissue lysate samples were diluted in lysis buffer to a final concentration of 1 mg/ml and then further diluted in a specific assay reagent. Matched colon cancer tissue and autologous reference tissue samples were analyzed in the same run, and each run included patients with different mutation status. Two assay quality controls were prepared in-house using reference colon tissue treated equally to the samples. The assay quality controls were included in each run to evaluate assay performance and to determine intra-assay coefficient of variation (CV%) and the intermediate precision which expresses within-laboratories variations: different days, different analysts, different reagent lots, etc. The mean intra-assay CV% was 8% and the mean intermediate precision was 20%. Limit of detection (LOD) was determined using 3 standard deviations from the background. The mean LOD was estimated to be 3.8 pg/ml for tERK, 3.6 for pERK, 10 pg/ml for tAKT, 2 pg/ml for pAKT and 3.2 pg/ml for cyclin d.

### Mutation analysis

The mutational statuses of PIK3CA, BRAF and KRAS mutations were investigated in the cancer tissues using droplet digital polymerase chain reaction (ddPCR). This method has been described in detail by CEB Thomsen et al.^12^. The most frequent KRAS and BRAF mutations were investigated (KRAS G12D, G12V and G13D and BRAF V600E). If the samples were negative for these mutations, they were analysed for 14 KRAS mutations in codons 12, 13, 61, 117 and 146 and 9 NRAS mutations in codons 12, 13 and 61. These 27 KRAS and NRAS mutations were selected based on the literature^6,13^ and cover mutations found in more than 0.2% of colorectal cancers. All samples were analysed for the four most common PIK3CA mutations (E542K, E545K, H1047R and H1047L).

### Statistical methods

Data were evaluated using NCSS software version 2019 (Kaysville, UT, USA). All data was found to be non-normally distributed and hence non- parametric statistic was used. Groups were compared using the Mann Whitney test for unpaired groups and Wilcoxon Rank Sum test for paired groups. For correlation analyses Spearman’s ρ were used. Overall survival (OS) was defined as the time from operation to death from any cause. Disease free survival (DFS) was defined as the time from operation to the first documented recurrence, locally or distant of colon cancer or death of any cause. Cut-offs giving the best statistics were used to separate the patients in subgroups. Survival analyses were carried out using Kaplan–Meier plots and survival differences for subgroups were compared using log-rank test. All reported P-values were two-sided and P < 0.05 were considered statistically significant.

## Results

### Autologous reference tissue and cancer tissue

**Figure 2:**
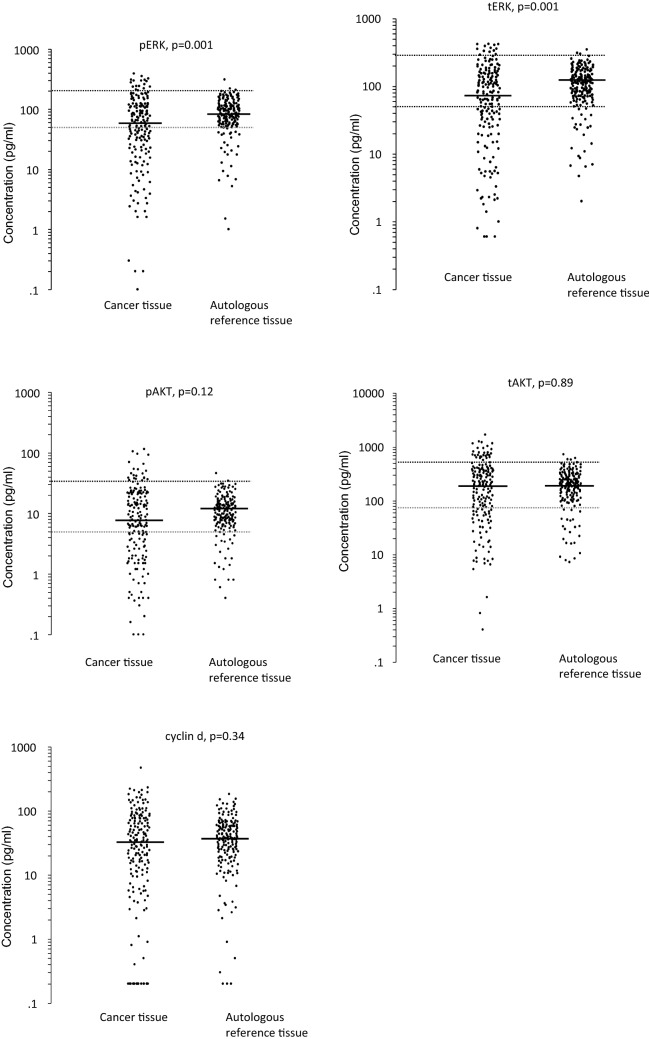

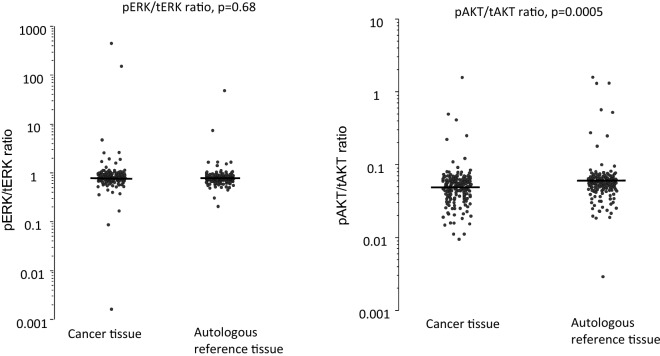
Pathway protein concentrations in autologous reference and cancer tissue. The black line demonstrates the median and the dotted line indicates the cut-off established from the autologous reference tissue.

tERK, pERK, tAKT, pAKT and cyclin d were measured in both autologous reference tissue and colon cancer tissue (Fig. 2; Table 2). Both pERK and tERK were found to be down-regulated in cancer tissue (p = 0.001), while pAKT, tAKT and cyclin d showed no differences between the tissues. The ratio of phosphorylated to total protein levels was also investigated and showed no differences between the tissues for ERK but a significant down-regulation in cancer tissue for AKT (p = 0.0005). The interrelationship between the pathway proteins was studied and significant correlations were found in both the autologous reference tissue and cancer tissue (Supplementary Information S2).

**Table 2:** Pathway protein concentrations in autologous reference and cancer tissue.

	Autologous reference tissue	Cut off values	Cancer tissue
tERK	111 (2–349)	< 50	75 (0.6–421)
		50–300	
		≥ 300	
pERK	83 (1–8174)	< 50	61 (0.1–391)
		50–200	
		≥ 200	
tAKT	202 (7–721)	< 70	199 (0.4–1674)
		70–500	
		≥ 500	
pAKT	11 (0.4–46)	< 5	8 (0.1–115)
		5–35	
		≥ 35	
Cyclin d	37 (0.2–183)	n.d.	33 (0.2–470)
pERK/tERK ratio	0.806 (0.21–50)	n.d.	0.802 (0.002–463)
pAKT/tAKT ratio	0.058 (0.003–1.58)	n.d.	0.051 (0.01–1.57)

All measurements are in pg/ml. The median and range are shown for each pathway protein. Cut-off values were established from the autologous tissue.

### Pathway proteins and mutational status

Of the 176 patients, 58 patients were Wt for all investigated mutations (33%). Patients with BRAF mutations (n = 47, 26.7%), KRAS mutations (n = 48, 27.3%), NRAS mutations (n = 4, 2.3%) and PIK3CA mutations (n = 4, 2.3%). Patients with mutual mutations for PIK3CA and BRAF (n = 6, 3.4%) or PIK3CA and KRAS (n = 8, 4.5%). One patient had no mutational data.

**Figure 3:**
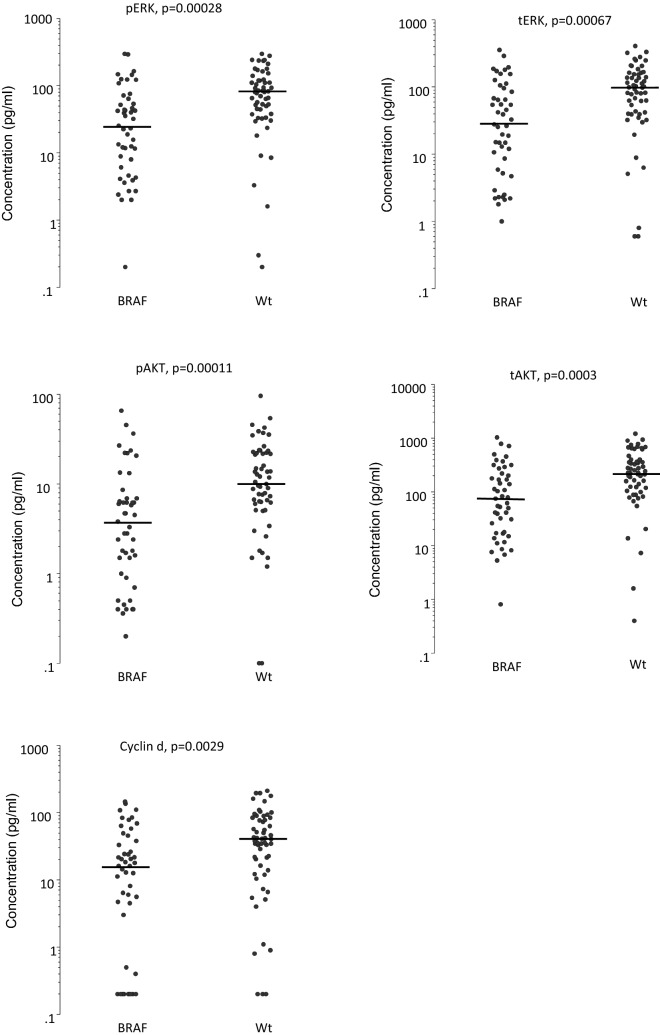
Pathway protein concentrations in cancer tissue with BRAF mutations or Wt.

The concentrations of tERK, pERK, tAKT, pAKT and cyclin d in colon cancer tissue were compared with the mutational statuses. The median and range values for all proteins are shown in Table 3. Patients with BRAF mutations had significantly lower concentrations of all investigated pathway proteins compared to Wt patients (Fig. 3): tERK (p = 0.0007), pERK (p = 0.0003), tAKT (p = 0.0003), pAKT (p = 0.0001) and cyclin d (p = 0.003). There were no significant differences between patients with KRAS mutations and Wt patients for either pathway protein. The ratio of phosphorylated to total protein levels showed no differences between patients with KRAS or BRAF mutations and Wt patients. Due to the limited number of patients, no statistical calculation was made for the remaining mutation variations.

**Table 3:** Pathway protein concentrations according to mutation status. The median and range are shown for each pathway protein. Wt is wildtype for all investigated mutations. All measurements are in pg/ml.

Cancer Tissue	Wt n = 58	KRAS n = 48	BRAF n = 47	PIK3CA n = 4	NRAS n = 4	KRAS and PIK3CA n = 8	BRAF and PIK3CAn = 6
tERK	99 (0.6–407)	111 (0.6–393)	28 (1–356)	40 (4.5–247)	293 (43–418)	69 (25–421)	25 (19–204)
pERK	81 (0.2–298)	85 (0.1–322)	25 (0.2–298)	35 (1.6–152)	212 (31–354)	57 (20–391)	23 (14–153)
tAKT	226 (0.4–1212)	298 (6.5–1158)	75(0.8–1030)	39 (7.6–294)	668 (353–1674)	109(60–1253)	90 (38–380)
pAKT	10 (0.1–96)	14 (0.1–93)	3.8 (0.2–66)	2.4 (0.2–22)	34 (8–115)	7 (3.7–106)	2.3 (0.7–24)
cyclin d	39 (0.2–211)	49 (0.2–231)	16 (0.2–146)	36 (2.9–76)	139(27–470)	40 (11–209)	11 (0.2–68)
pERK/tERK ratio	0.81 (0.002–463)	0.80 (0.17–158)	0.84 (0.087–2.7)	0.71 (0.36–1.77)	0.76 (0.70–0.85)	0.78 (0.59–0.96)	0.75 (0.73–0.92)
pAKT/tAKT ratio	0.05 (0.01–0.41)	0.05 (0.01–0.12)	0.05 (0.02–1.57)	0.06 (0.02–0.07)	0.05 (0.02–0.07)	0.06 (0.05–0.08)	0.02 (0.01–0.07)

### Clinical data

For each pathway protein, the median concentration value in cancer tissue was used as the cut-off and tested for its ability to distinguish between patients with different prognoses. Moreover, the autologous reference tissue was used to establish cut-off values for pERK, tERK, pAKT and tAKT (Fig. 2; Table 2). No significant differences were found between patients with high concentrations and those with low concentrations using either discrimination cut-off regarding disease free survival or overall survival.

**Figure 4:**
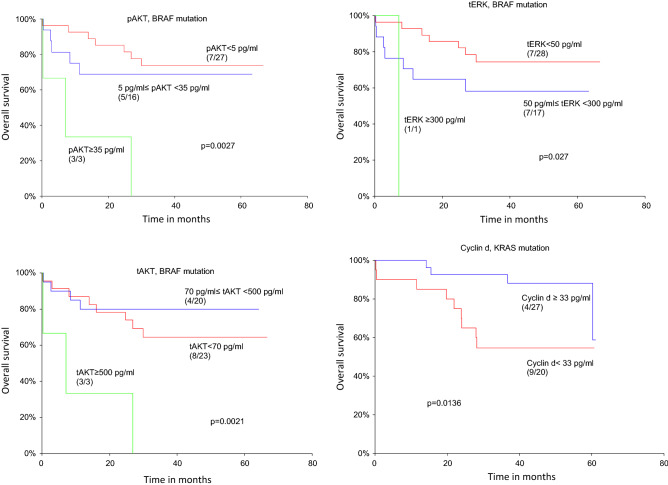
Overall survival in patients with BRAF or KRAS mutations. Kaplan–Meier curves. Numbers in parentheses indicate events/total number of patients.

Using the cut-off values established from the autologous reference tissue and categorizing the cohort according to BRAF mutations a decreased overall survival was observed for patients with high levels of tERK (p = 0.027), tAKT (p = 0.0021) and pAKT (p = 0.0027) (Fig. 4). Moreover, patients with BRAF mutations showed decreased disease-free survival for pERK (p = 0.044), tAKT (p = 0.029) and pAKT (p = 0.050) (Supplementary Information S3). Using the median as the cut-off resulted in no significant differences in patients with BRAF mutations however patients with KRAS mutations and low levels of cyclin d demonstrated decreased overall survival (p = 0.0136) (Fig. 4).

## Discussion

This study demonstrates a strong correlation between intracellular signaling protein concentrations in colon cancer tissue and mutational BRAF status and significantly prolonged overall survival for patients with BRAF mutations and low levels of AKT or ERK.

To our knowledge, this is the first study to measure the signaling proteins AKT, ERK and cyclin d in colon cancer tissue using quantitative methods, which we developed using Simoa technology.

We found that colon cancer tissues that had BRAF but not KRAS mutations demonstrated significantly lower concentrations of total ERK, AKT, cyclin d and activated ERK and AKT as compared to Wt. Most studies investigating intracellular signaling proteins in relation to BRAF mutation status use IHC and include a low number of patients^14–16^. One study though with a large cohort of CRC patients found that patients with BRAF mutations had increased nuclear and cytoplasmic pERK IHC staining as compared to Wt^17^. This is not in line with our study and may be due to the diverse analytical methods and patient cohorts. A study by Baba et al. found no differences in pAKT IHC expression in 109 CRC patients with BRAF mutations as compared with Wt^18^. Wan et al. found no differences in ERK between KRAS mutation and Wt CRC patients^19^, which is in agreement with our finding.

Cancer tissues with genetic mutations might result in the constitutive activation of intracellular pathways. It is therefore plausible that an increased activation and turn-over may lead to over-production or consumption of intracellular signaling proteins as seen in patients with BRAF mutations. However, we still lack an explanation as to why patients with KRAS mutations demonstrate pathway protein concentrations similar to those of Wt patients but not BRAF-mutated patients. Though this simple model for activation implies decrease and change in pathway proteins, as evidenced in patients with BRAF mutations, the mechanism behind could be more complex^1,2,20^.

No statistically significant correlations were found between ERK, AKT or cyclin d and disease-free or overall survival in the localized colon cancer patient cohort used in this study. However, stratification by mutation status showed that patients with BRAF mutations and high concentrations of ERK or AKT had low overall survival. As these results are based on a limited number of patients in each group, more patients are needed to support these findings.

Studies on AKT or ERK activation have yielded variable results regarding survival. Malinowsky et al. showed that activation of AKT correlated with decreased survival, while Baba et al. showed that AKT activation was associated with a favorable outcome. Schmitz et al. found that the activation of ERK but not AKT predicted poor prognosis^16,18,21^. The majority of these studies included both colon- and rectal tumors, and differences in prognostic value in the two groups may be possible. The divergent results among these studies may also be due to differences in methods such as mutation data and sample sizes.

This study found a statistically significant correlation between pathway protein concentrations and mutational status. However, the change in pathway protein concentrations is too small to be used as a screening indicator of mutations in clinical practice.

As known from the complement and coagulation pathways, the correct way to detect an increase in activity may be to quantify not the native proteins but degradation or split products from the single intracellular pathway proteins. Therefore, we now aim to develop specific antibodies and methods for measuring these degradation products as previously done for complement C3d^22,23^. We assume this approach will increase both the ability to predict mutations and survival.

## Supplementary information

Supplementary Information.

## Data Availability

The dataset contains person-sensitive data that were used under license for the study. Thus, the data are not publicly available. Upon reasonable request and with permission from the relevant legal authorities under existing laws, the data may be made available by the authors.

## Abbreviations

Simoa: Single molecule array
SβG: Streptavidin-β-galactosidase
RGP: Resorufin-β-d-galactopyranoside
AEB: Average number of enzymes per bead
