# Supplementary material for: Decreased concentrations of intracellular signaling proteins in colon cancer patients with BRAF mutations
Source: Sci Rep. 2020 Nov 18;10:20113. doi: 10.1038/s41598-020-77109-8 (PMC7675974; doi:10.1038/s41598-020-77109-8)
Supplement: Supplementary file 1 — Supplementary Information. [file 41598_2020_77109_MOESM1_ESM.docx]

**Decreased concentrations of intracellular signaling proteins in colon cancer patients with BRAF mutations**

Dorte Aa. Olsen^1,3,4*^, Caroline EB. Thomsen^2,3,4^, Rikke F. Andersen^1,3,4^, Jonna S. Madsen^1,3,4^, Anders Jakobsen^2,3,4^ and Ivan Brandslund^1,3,4^

^1^Department of Biochemistry and Immunology, Lillebaelt Hospital, University Hospital of Southern Denmark, Vejle, Denmark.

^2^Department of Oncology, Lillebaelt Hospital, University Hospital of Southern Denmark, Vejle, Denmark.

^3^Department of Regional Health Research, University of Southern Denmark, Odense, Denmark.

^4^Danish Colorectal Cancer Center South, Lillebaelt Hospital, University Hospital of Southern Denmark, Vejle, Denmark.

* Department of Biochemistry and Immunology

Lillebaelt Hospital

Beriderbakken 4

DK – 7100 Vejle

Phone: +45 79406633

E-mail: [dorte.aalund.olsen@rsyd.dk](mailto:dorte.aalund.olsen@rsyd.dk)

Short title: Pathway proteins in colon cancer

**Supplementary information**

**S1**

The methods were developed as single-plex assays using a Simoa 2-step assay for tAKT and pAKT and a 3-step assay for tERK, pERK, and cyclin d. Before running, the following reagents were prepared: capture beads (1/3) were mixed with helper beads (2/3) in bead diluent buffer (Quanterix) and diluted to a final concentration of 2.0*10^7^ beads/ml. The biotinylated detector antibodies were diluted in sample/detector diluent (Quanterix) to final concentrations of 0.2 mg/L for tAKT and pAKT, 0.1 mg/L for tERK, pERK and cyclin d. The SβG was diluted in SβG diluent (Quanterix) to 150 pM. After the prepared reagents and consumables were loaded, 8 calibrators for each assays were prepared in diluent A (Quanterix) ranging from 0 to 2000 pg/ml for pAKT, 0 to 3000 pg/ml for cyclin d and 0 to 5000 pg/ml for tAKT, tERK and pERK. The samples and controls were diluted 30-fold in diluent A and loaded onto the instrument in a 96-well microtiter plate. The calibrators and the controls were run in duplicates and the samples were single determinations. The following steps were performed by the instrument. For the 2-step assay, 25 µl of capture bead was pipetted into a cuvette together with 100 µl of sample, control or calibrator and 50 µl of biotinylated detection antibody. An incubation step was performed for 30 minutes and the beads were then magnetically separated and washed. For the 3-step assay, 25 µl of capture bead was pipetted into a cuvette together with 100 µl of sample, control or calibrator and incubated for 40 minutes. The beads were then washed and 100 µl of detection antibody was added, and an incubation step was performed for 5 minutes followed by washing the beads. The following steps were identical for both the 2-step and 3-step assays. 100 µl of SβG was added to the cuvette by the instrument and an incubation step was performed for 5 minutes. The beads were then separated magnetically and washed following the addition of RGP substrate. The bead substrate mixture was then loaded onto the Simoa disc containing an array of 216,000 micro-wells and sealed with oil. If protein had been captured and labeled, the SβG hydrolyze the RGP substrate into a fluorescent product that can be measured. At low concentrations of proteins, beads carry either zero or low numbers of enzymes and protein concentration is quantified by counting the presence of “on” or “off” bead (digital). At higher concentration of protein, each bead carries multiple enzymes and the total fluorescence signal is proportional to the amount of protein in the sample (analog). Both the digital and analog calculations use the unit “average number of enzymes per bead (AEB)”. The concentrations of protein in the unknown samples were interpolated from the calibrator curves obtained by 4-parameter logistic regression fitting.

**S2. Correlations between the pathway proteins.**

| The Spearman’s rank  correlation coefficients | pERK | tAKT | pAKT | cyclin d |
| --- | --- | --- | --- | --- |
| Cancer tissue |  |  |  |  |
| tERK | 0.91 | 0.80 | 0.85 | 0.81 |
| pERK | - | 0.85 | 0.90 | 0.86 |
| tAKT | - | - | 0.93 | 0.81 |
| pAKT | - | - | - | 0.88 |
| Autologous reference tissue |  |  |  |  |
| tERK | 0.93 | 0.74 | 0.74 | 0.69 |
| pERK | - | 0.75 | 0.76 | 0.67 |
| tAKT | - | - | 0.78 | 0.58 |
| pAKT | - | - | - | 0.66 |

The Spearman’s rank correlation coefficients are shown.

**S3. Disease free survival in patients with BRAF mutations.**

Kaplan-Meier curves. Numbers in parentheses indicate events/total number of patients.

0

10

20

30

40

50

60

0%

20%

40%

60%

80%

100%

Disease free survival

Time in month

p=0.05

pAKT, BRAF mutation

pAKT≥35 pg/ml

(1/3)

5 pg/ml≤ pAKT <35 pg/ml

(2/16)

pAKT<5 pg/ml

(6/27)

0

10

20

30

40

50

60

0%

20%

40%

60%

80%

100%

Time in month

Disease free survival

tAKT, BRAF mutation

p=0.029

tAKT≥500 pg/ml

(1/3)

tAKT<70 pg/ml

(6/23)

70 pg/ml≤ tAKT <500 pg/ml

(2/20)

0

10

20

30

40

50

60

0%

20%

40%

60%

80%

100%

Disease free survival

Time in month

pERK, BRAF mutation

p=0.044

pERK≥200 pg/ml

(1/2)

50 pg/ml≤ pERK <200 pg/ml

(1/12)

pERK<50 pg/ml

(7/32)
